# DNA Persistence in a Sink Drain Environment

**DOI:** 10.1371/journal.pone.0134798

**Published:** 2015-07-31

**Authors:** Eric M. Winder, George T. Bonheyo

**Affiliations:** Pacific Northwest National Laboratory, Sequim, Washington, United States of America; Naval Research Laboratory, UNITED STATES

## Abstract

Biofilms are organized structures composed mainly of cells and extracellular polymeric substances produced by the constituent microorganisms. Ubiquitous in nature, biofilms have an innate ability to capture and retain passing material and may therefore act as natural collectors of contaminants or signatures of upstream activities. To determine the persistence and detectability of DNA passing through a sink drain environment, *Bacillus anthracis* strain Ames35 was cultured (6.35 x 10^7^ CFU/mL), sterilized, and disposed of by addition to a sink drain apparatus with an established biofilm. The sink drain apparatus was sampled before and for several days after the addition of the sterilized *B*. *anthracis* culture to detect the presence of *B*. *anthracis* DNA. Multiple PCR primer pairs were used to screen for chromosomal and plasmid DNA with primers targeting shorter sequences showing greater amplification efficiency and success. PCR amplification and detection of target sequences indicate persistence of chromosomal DNA and plasmid DNA in the biofilm for 5 or more and 14 or more days, respectively.

## Introduction

Biofilms are found in most environments, particularly at the interfaces of solid, liquid, and atmospheric environments, and may range in appearance from the dry desert varnish found on rock surfaces in arid environments to the more familiar “slimes” including periphyton found in aquatic environments. The structure of a biofilm allows microorganisms to form a distinct microenvironment that is separate from, yet interactive with the surrounding environment [1]. A critical feature is the matrix of extracellular polymeric substances (EPS) secreted by cells that reside in the biofilm. The EPS is composed of a heterogeneous mixture of polysaccharides, lipids, nucleic acids, and proteins [2]. Each component of the EPS plays a particular role in providing protection, sorption capabilities, nutrient/waste processing, and/or planktonic release of progeny cells [3]. Of particular interest, for this study, is the molecular sieve capability of an established biofilm that allows it to capture, sort, and sequester particular molecules from passing water [4], a sort of sticky trap (cf. flypaper) for passing cells, biomolecules, and metals. This collection potential has several implications, including the extent to which biofilms may create and preserve a historical record of passing chemistry.

Little is currently known about the accumulation and retention properties of environmental biofilms, except that they allow microorganisms to survive and prosper even in nutrient-limited, dynamic, or hazardous environments. External DNA (eDNA) is an integral part of many biofilms that provides structural support and, in the case of the microorganism *Pseudomonas aeruginosa*, is a requirement for biofilm formation and permanence [5]. The addition of the DNA cleaving enzyme, DNase I, was shown to prevent biofilm formation and cause the dissolution of an established biofilm [6]. Although many microorganisms, including those in established biofilms, actively release extracellular enzymes to breakdown macromolecules into smaller, more easily assimilated materials [7], the dependence of eDNA for biofilm structural support suggests that DNA trapped from the surrounding aqueous environment may be retained by a biofilm for an extended period of time. DNA can remain intact for longer than 50,000 years in an ideal, stable aqueous environment [8]; however, the dynamic hydration state and chemistry of a sink drain biofilm environment may not be ideal for the preservation of DNA.

Sink drains have been previously investigated for their ability to harbor a particular community of microorganisms [9], to examine the diversity of drain communities [10], and to examine the potential emergence of resistance to antimicrobial agents found in wastewater [11]; however, the capability of biofilms to retain genetic signatures from the surrounding environment has not previously been studied. To better understand the dynamics of waste-stream DNA retention by biofilms, this project utilized laboratory sink drain biofilms and sterilized bacterial cultures as a practical model. The sterilized cultures provide DNA that is still located within the cell bodies and DNA that has been released from ruptured or fragmented cells. As our interest was to determine the ability of biofilms to retain any genetic signature from the waste stream, we made no effort in this study to differentiate between the preservation of intracellular or extracellular DNA.

## Materials and Methods

### Biofilm Establishment

**Fig 1:**
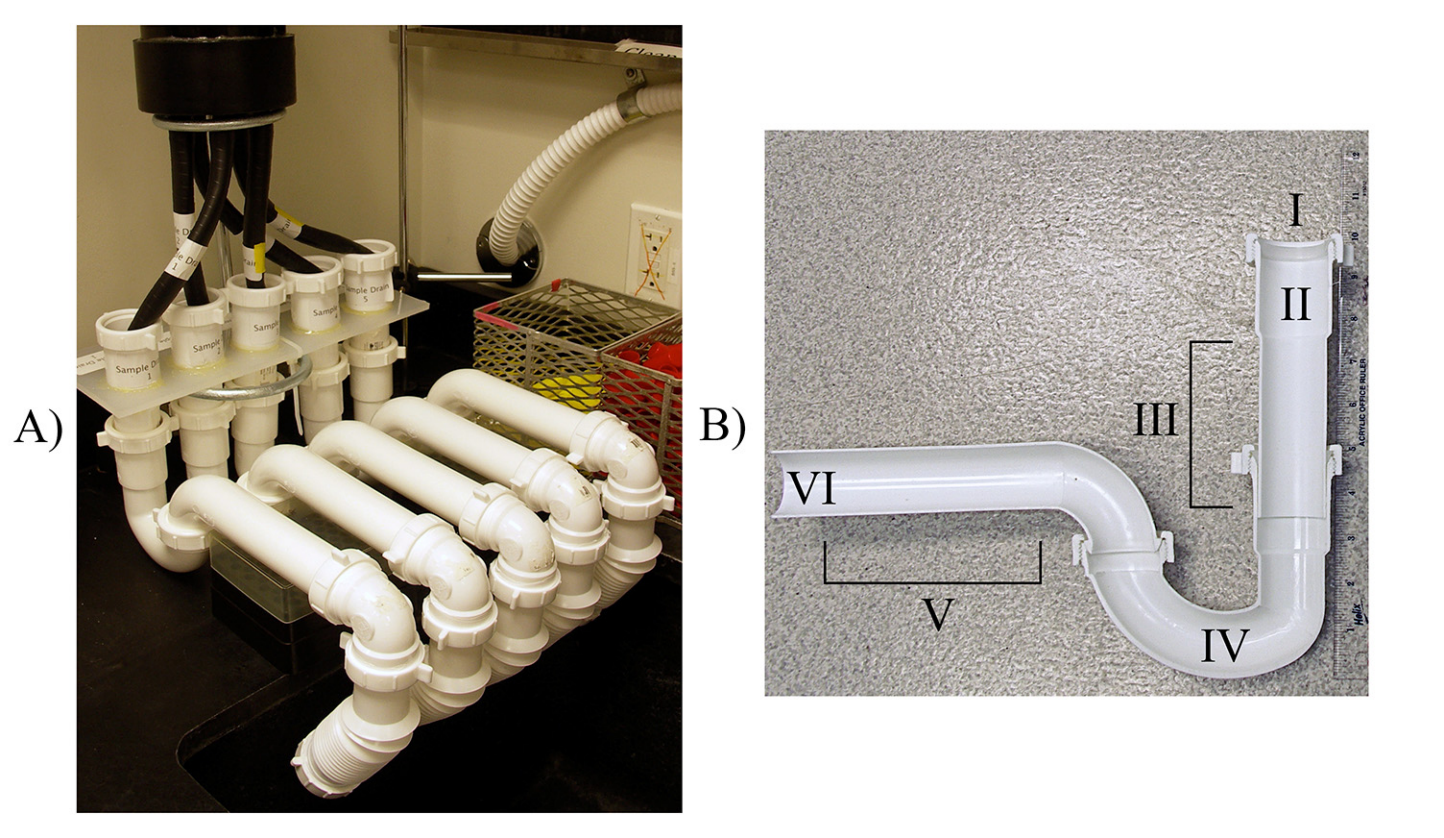
A) Sink drain apparatus for establishment and maintenance of experimental biofilms and B) Cross-section and identification of the different sections of a sink drain; I, sink drain connection; II, tailpiece; III, tailpiece extension; IV, p-trap; V, p-trap extension; VI, out pipe to sewer.

A set of 5 identical sink drains were connected to a shared source input to assure that each drain was exposed to identical fluid streams (Fig 1A). Because sample collection disturbs and removes sections of the biofilm, the replicate drains were necessary to allow us to collect multiple samples over time and from equivalent biofilms. The sink drain apparatus was built using standard polypropylene-based 1-1/2” sink drain plumbing with each drain having a tailpiece, p-trap, and p-trap extension (Fig 1B). Initial seeding of the biofilm for the drain system was accomplished by physical removal of biofilm from an existing laboratory p-trap, vortexing to homogenize, and subsequently adding a fraction of this biofilm mixture to each of the 5 test drains. This biofilm material was allowed to establish in the test drains over a period of 2 months with weekly additions of Milli-Q (18 MΩ ultrapure) H_2_O to replenish evaporative losses and bi-weekly nutrient supplementation by addition of 4 mL nutrient broth and 4 mL potato dextrose broth to promote the growth of microorganisms.

### Cell Culturing

*Bacillus anthracis* strain Ames35 (BEI# NR-10355) was cultured in 500 mL aliquots of Luria broth (LB) medium for 36 hours at 37°C at 125 rpm. A 100 μL sample of the culture was removed for serial dilution and plating to determine the number of viable colony forming units (CFU) on LB agar plates (incubated at 37°C). The remaining culture was split into two equal fractions with one fraction sterilized by addition of household bleach (final concentration equal to 10%), while the other fraction was sterilized using an autoclave (121°C for 15 minutes). Three 1 mL aliquots were removed from both sterile fractions and stored at -85°C for reference analysis. The remaining ~1.4 L of sterilized material was poured into to the inlet reservoir and distributed amongst the five sink drains. This material was allowed to sit in the apparatus for 24 hours at which time 2 Liters of 50/50 (v/v) mix of Milli-Q H_2_O and hot tap water was added to the inlet reservoir. This 2 Liter flush was repeated approximately every 24 hours, for the length of the experiment to simulate periodic use of the drain system. Additionally, all staff using the adjacent sink collected and recorded the volume and contents of all wastewater each day, which was also disposed of down the drain system.

### Biofilm Sampling

**Fig 2:**
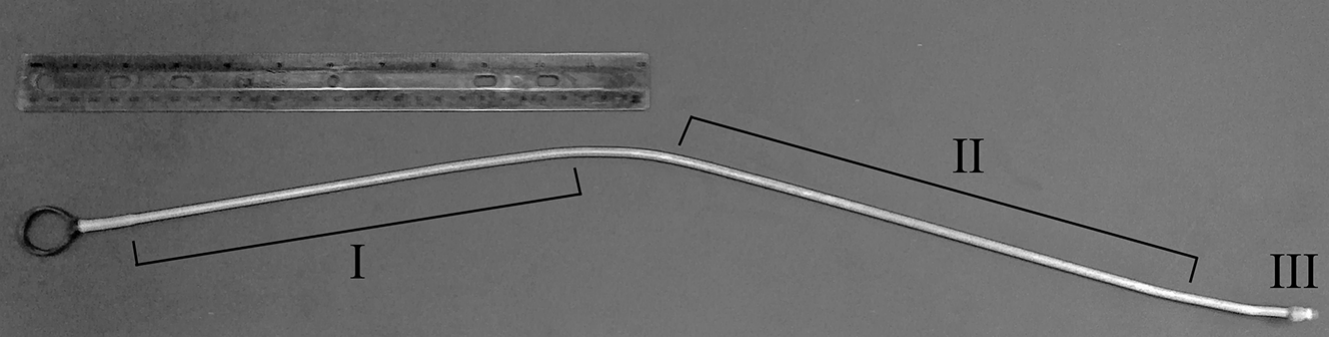
Custom designed sampling device with PVC coating for chemical and heat resistance, rigid support (I), flexible shaft to sample past the p-trap (II), and male bolt end (III) to attach various sampling tips.

**Fig 3:**
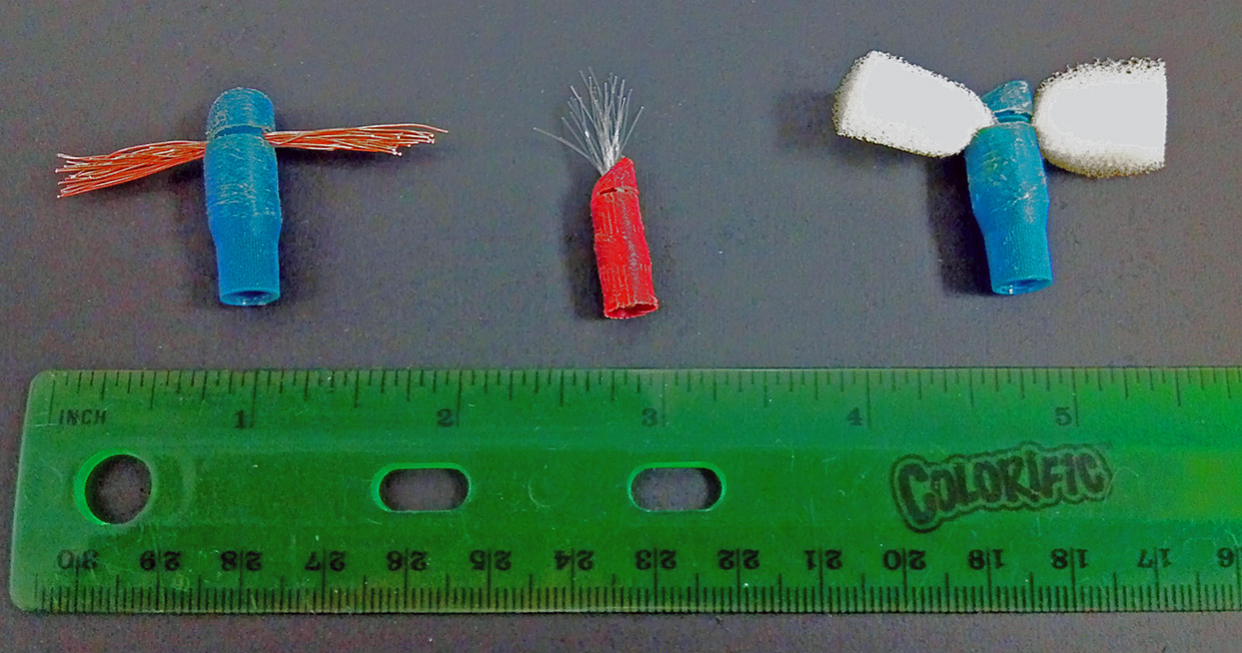
Sampling tips with female tap ends to attach to the tip of the sampling device. Various sample materials shown, from left to right: copper wire, nylon fibers, and polyether foam.

A sample of biofilm was collected from one of the 5 drains prior to adding the sterile cell culture material and again prior to each daily flush with water. At each sampling time point, the first sample was obtained utilizing a sterile synthetic Dacron swab (Copan Diagnostics Incorporated, Murrieta, CA) to sample the tailpiece of the sink drain, after which the tip was removed and placed in 1 mL of nuclease-free H_2_O. Subsequently, a custom made sampling device was used to sample the p-trap and p-trap extension using both a foam-insert tip (Figs 2 and 3), and a copper-insert tip (Fig 2). The sampling device was sterilized by submersion in 10% household bleach for a minimum of 10 minutes. The sampling tips were soaked in DNAzap (Ambion, Grand Island, NY), rinsed using 18 MΩ Milli-Q H_2_O, sterilized by autoclaving (121°C for 15 minutes) and finally allowed to dry at 60°C. When collecting samples with the foam tip, the tip was allowed to absorb fluids for 30 seconds, in the p-trap, prior to sampling throughout the remainder of the drain. After sampling, the tips were first rinsed with 10 mL of nuclease-free H_2_O, in a 50 mL falcon tube, to retrieve any loosely associated material, and then the tips were removed from the sampling device and placed in the same vial of nuclease-free H_2_O that was used for rinsing. Finally, a 10 mL sterile, disposable, graduated pipette was inserted into the drain system to obtain 5 mL of soluble/suspended p-trap material. All sample material was stored at -85°C.

### DNA Isolation

DNA was isolated from the samples by first vortexing each sample in a pulsating on/off pattern for 30 seconds, followed by centrifugation at 16,100 x *g* for 90 minutes at 4°C. After centrifugation the pelleted material was removed by pipette with minimal supernatant carryover. The transferred pellet sample was then split into two fractions. DNA from the first fraction was isolated using the MOBIO DNA Clean-up Kit (Carlsbad, CA) following manufacturer protocols with a final elution volume of 50 μL, while DNA from the second fraction was isolated using the MOBIO PowerSoil DNA Kit with final elution volume of 100 μL. While both DNA isolation methods used silica column binding as their final step, both chemical and mechanical lysis were utilized upstream to reduce DNA isolation bias that could ultimately produce false negatives in the results. The two DNA preparations from each sample were then combined and used as template DNA for PCR.

### Polymerase Chain Reaction and DNA Fragment Visualization

Multiple primer sets detailed in Table 1 below were utilized to amplify specific regions of DNA from *B*. *anthracis* using 5 μL of template DNA in 25 μL reactions with 2.5 units of GoTaq Flexi DNA Polymerase (Promega, Madison, WI), 0.5 μM final concentration primers (Integrated DNA Technologies, Inc., Coralville, IA) and 3.5 MgCl_2_ (Promega) final concentration All amplifications were performed using an Eppendorf MasterCycler Gradient Thermocycler (Eppendorf AG, Hamburg, Germany) with 95°C initial 5 minute denaturation, 35 cycles of (1 minute at 94°C, 1 minute at 47°C, 2 minutes at 72°C), and a final 5 minute extension at 72°C (45 minute final extension for universal eubacteria primers). Following PCR amplification, 10 μL of each sample was loaded into a 3% agarose gel, separated by electrophoresis, stained with ethidium bromide, and visualized utilizing a Syngene U:Genius (Synoptics Ltd, Cambridge, England) gel imager with ultra-violet illumination. Alternatively, 2 μL of sample was added to 18 μL of 1X PCR buffer and loaded onto LabChip GX digital capillary gel electrophoresis system (PerkinElmer, Waltham, MA).

**Table 1:** Primers utilized for *Bacillus anthracis* & eubacteria DNA amplification and the expected amplicons.

*Bacillus anthracis* primers	Expected Amplicon Size(s) (bp)	Primer Target	Specificity
**ITSeub [16]**	225, 232, 459	Ribosomal Intergenic Spacer Region	eubacteria
**BA813 [17]**	168	Chromosomal marker BA-813	*B*. *anthracis*
**BA-5449 [18]**	1017	Chromosomal marker BA-5449	*B*. *anthracis*
**PA7/6 [19]**	211	pXO1 Protective Antigen Gene	*B*. *anthracis*
***cya*-1 [20]**	721	pXO1 Edema Factor Gene	*B*. *anthracis*

### Gel Extraction and DNA Sequencing

Amplified DNA was isolated from agarose gel slices utilizing the Wizard SV Gel and PCR Clean-Up System (Promega, Madison, WI) following the manufacturer’s protocol. Extracted DNA was cloned using the pGEM-T Easy Vector System (Promega, Madison, WI) following the manufacturer’s suggested protocol for ligation and chemical transformation of *Escherichia coli* strain DH5α. Cultures were incubated at 37°C and frozen cell pellets were sent to Beckman Coulter Genomics (Danvers, MA) for Sanger sequencing.

## Results

**Fig 4:**
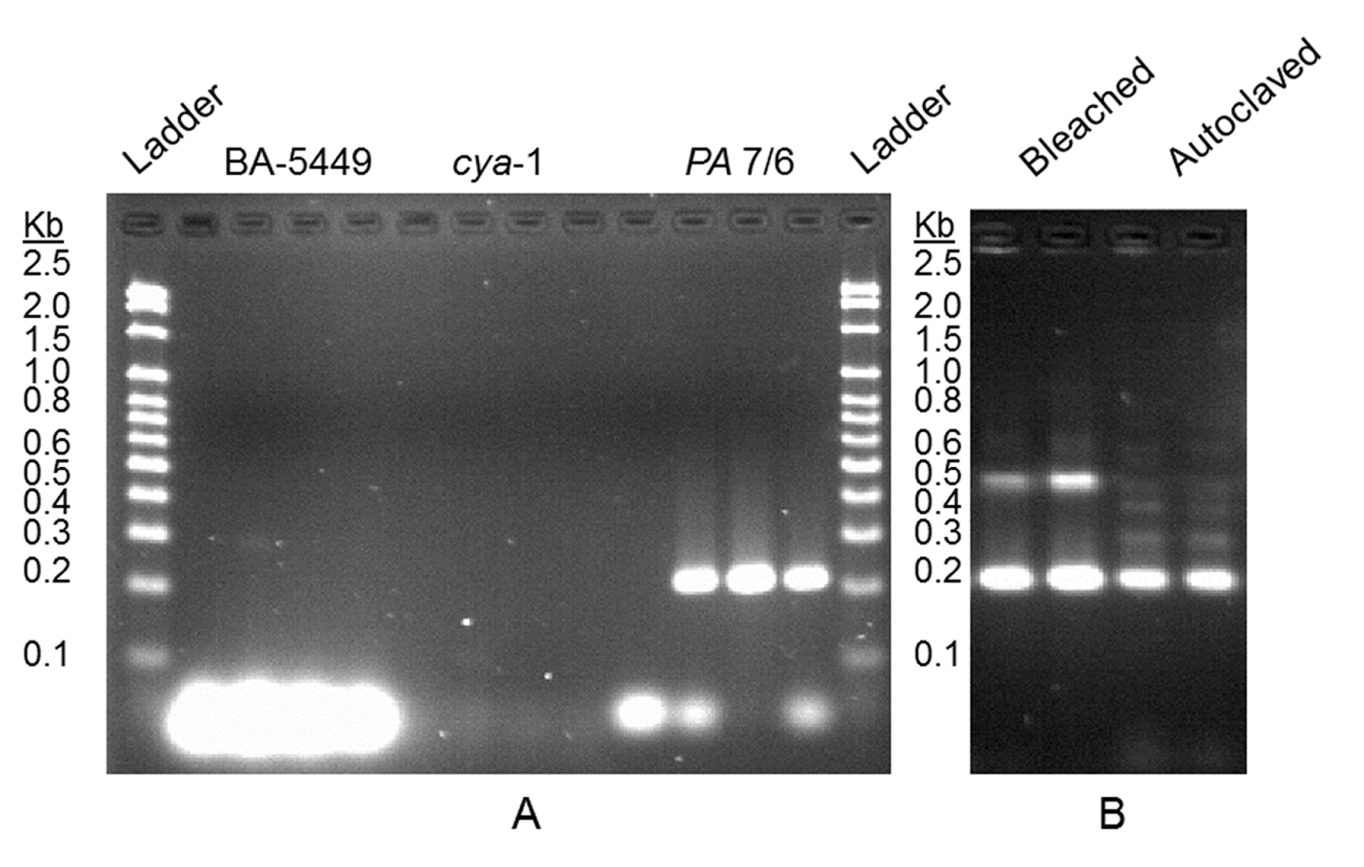
Agarose gel images for *Bacillus anthracis* PCR samples. Depicted in A, from left to right, is 100 bp ladder, no-template control (NTC) and 3 reactions utilizing BA-5449 primer set, NTC and 3 reactions utilizing *cya*-1 primer set, NTC and 3 reactions utilizing *PA*7/6 primer set and 100 bp ladder. Depicted in B, from left to right, are duplicate reactions with bleached *Bacillus anthracis* template material and duplicate reactions with autoclaved *Bacillus anthracis* template material, with all 4 reactions utilizing the ITSeub primer pair.

The PA7/6 primer set targeting the protective antigen gene on the *Bacillus anthracis* plasmid X01 yielded the expected amplification band of 211 bp. The ITSeub primer pair targeting the ten chromosomal ribosomal intergenic spacer regions of *B*. *anthracis* yielded multiple bands in addition to the three expected sizes for *B*. *anthracis*. The cya-1 and BA-5449 primer sets targeting the edema factor of pXO1 and chromosomal marker BA-5449, respectively, showed no amplification bands (Fig 4A). The three samples tested for each primer pair include: an autoclaved *B*. *anthracis* control DNA sample, the day 1 bleached/autoclaved sink drain apparatus inoculum.

Due to the inability to efficiently amplify DNA from *B*. *anthracis* utilizing the BA-5449 and *cya*-1 primers, coupled with the high amplification for the PA 7/6 and ITSeub a new primer, BA813, was selected for *B*. *anthracis* chromosomal DNA amplification.

**Fig 5:**
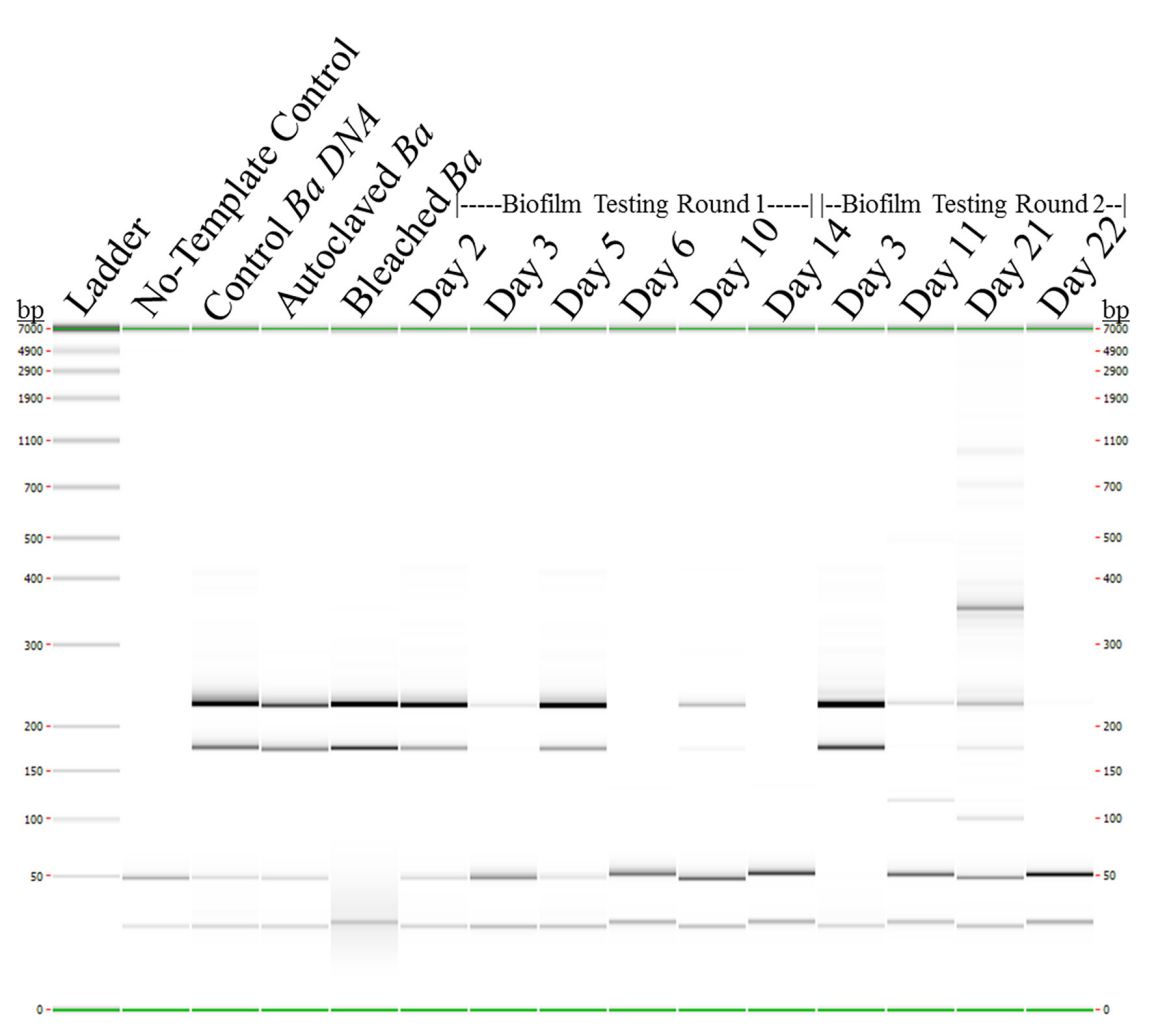
Digital capillary electrophoresis gel image from LabChip GX acquired using HT DNA Hi Sens(itivity) Reagent Kit. Sample wells contain, from left to right: LabChip GX Ladder, No-Template Control, Control Isolated *Bacillus anthracis* strain Ames35 DNA, Day 1 Autoclaved Sample prior to disposal, Day 1 Bleached Sample prior to disposal, 6-round 1 testing samples (Day 2, Day 3, Day 5, Day 6, Day 10 & Day 14 Samples.), and 4-round 2 testing samples (Day 3, Day 11, Day 21 & Day 22 Samples). Duplicate PCR amplification sample data for each time point obtained, but not shown.

The BA813, and PA 7/6 primer pairs were combined for multiplex amplification of foam-extracted samples from the sink drain apparatus. The *B*. *anthracis* strain Ames35 was cultured, sterilized and then disposed of down the sink drain apparatus system on two separate occasions. Multi-plex PCR results for samples collected over 36 days (14 days for round 1 and 22 days for round 2) resulted in the amplification profiles shown in Fig 5 below. No bands, as expected, are apparent in the BA813 and PA 7/6 no-template control (NTC) sample. The predicted 168 bp band expected for BA813 primer chromosomal amplification is observed in all samples through day 10 with the exception of the day 6 sample and the predicted 211 bp band amplified using the PA 7/6 primer pair showed the same amplification profile except that the band can be seen in day 14 pipette-extracted sample, data not shown.

Of note, after the day 5 sample was collected, the system was inundated with 18 liters of a bleach-killed (10% household bleach) diatom culture. This 18 liters of spent, bleached culture media then sat in the drain for 24 hours before day 6 sampling and 2-liter flush of the sink drain apparatus. The *Bacillus anthracis* target sequence may be present in the day 6 sample; however, further testing or sample concentration/separation may be required to obtain efficient amplification.

## Discussion

The targeted PCR amplifications demonstrate that DNA derived from a sterilized *B*. *anthracis* cell culture can be recovered from sink drain biofilms. The DNA post-autoclaving and/or bleach treatment is apparently highly degraded and the amplification of comparatively longer DNA targets is inefficient as shown in Fig 4A, where there is no amplification of the 1017 bp chromosomal target nor the 721 bp pXO1 targets, but amplification of the 211 bp pXO1 target is successful. This indicates a need for smaller target DNA sequences when working with DNA that may be highly degraded, much like the development and use of mini-STRs in human forensic science [12].

Qualitatively, the method of sterilization also has a great impact on the resultant targetable DNA. Utilizing the universal ITS primer set to compare DNA post-bleaching and post-autoclaving resulted in the amplification profile shown in Fig 4B. There does seem to be some degree of non-specific binding and/or jumping PCR [13] using this universal ITS primer pair, but looking directly for the expected target amplicon bands it can be noted that the 452 bp amplicon was easily amplified from the bleached cellular culture along with to the 225/232 bp target, while only the 225/232 bp target was amplified from the autoclaved material.

The loss of the 452 bp amplicon may result from enzymatic degradation during the autoclave process [14] and/or may also indicate an effect of copy number on the amplification and detection of a target DNA sequence by PCR. The *B*. *anthracis* genome has 10 ITS regions that may be amplified using the ITSeub primer pair: eight result in a 232 bp amplicon, one yields a 225 bp amplicon, and the remaining region yields a 452 bp amplicon. The two smaller amplicons are not readily resolved on our agarose gel nor LabChip System due to resolution restrictions. The combination of low copy number and increased likelihood of degradation preventing amplification of longer fragments of DNA should result in the loss of amplification efficiency for the 452 bp ITS region, as shown. This is in agreement with the results obtained for DNA persistence in the sink drain of chromosomal DNA versus plasmid as the plasmid DNA showed amplification over a greater period of time and the average copy number of the *B*. *anthracis* pXO1 plasmid has been shown to be three [15]. If the amplification was based solely on the size of the amplicon rather than copy number, the larger amplicon (211 bp) indicative of the pXO1 plasmid should have been lost first over the 168 bp chromosomal target.

The digital gel (Fig 5, above) shows good PCR amplification of the *B*. *anthracis* PA7/6 plasmid marker 211 bp target through day 10, with a temporary loss of the target signal on day 6. The 18 L of bleached diatom culture disposed of between days 5 and 6 would have been sitting undiluted in the drain system when the day 6 sample was collected, resulting in potential chemical inhibition for DNA isolation or competitive inhibition of PCR amplification of *B*. *anthracis* at that time point due the high amount of diatom DNA template. The diatom culture would have been diluted by the daily 2-liter flushes with water on days 6–9 prior to samples being collected on day 10. Indeed, the *B*. *anthracis* chromosomal/plasmid PCR amplicons reappeared on day 10 and the plasmid DNA was also amplified in the final sample collected on day 14 (pipette data not shown). This result is similar to that for biofilm testing round two, which indicates persistence of the plasmid and chromosomal targets through day 21.

The additional higher molecular weight band on day 21 at 355 bp of round two testing shows just how complex the biofilm sink drain environment can be and the richness of information that can be acquired. This higher molecular weight band along with the expected band fragments were excised from an agarose gel and processed for sequencing. The resultant data confirmed the 168 and 211 bp fragments as *B*. *anthracis* DNA, while the higher molecular weight band was an amplified region of an *E*. *coli* expression vector. This result leads us to hypothesize that we can discriminate the activity such as: synthetic biology and genetic engineering occurring in the built environment over another dissimilar activity by looking at the microbiome of and signatures retained in the sink drain biofilm.

The persistence of the target DNA in the sink drain environment after disposal and subsequent flushes (disappearance data not shown) indicates that DNA disposed of down the sink drain is retained by the established biofilm rather than remaining in the aqueous drain fraction. Although samples collected by pipette appeared to provide more target DNA, this is likely due to the pipette retrieving biofilm that had been dislodged into the aqueous phase during sampling with the foam and copper tipped sampling device.

## Conclusion

We have demonstrated that it is possible to detect DNA passing through a sink drain as part of a waste disposal event by sampling the previously established biofilm. Bleached and/or autoclaved killed microorganisms disposed of down a sink drain may be detected for an extended period of time post-disposal due to the retention of discarded culture DNA by the established sink drain biofilm. While the DNA retention mechanism and reasoning thereof in the biofilm is unknown, the retained DNA could have use as a structural element for the biofilm, carbon/nitrogen source or assimilated by the bacteria for transformation. Successful DNA detection requires the use of carefully designed oligonucleotide primers and, due to degradation of the sample during sterilization and subsequent retention in the sink drain biofilm, greater success is achieved by using primers pairs targeting smaller amplicons. Qualitatively, from these and other tests the probability of a positive DNA retention and detection event was determined more by the amount/availability of biofilm to sample rather than the length of time from disposal.

Further testing is planned to determine if there is variability in sink drain microbiome communities based-upon the activities undertaken in the built environment, and if this potential variability alters the retention characteristics of the biofilm for DNA or other passing chemicals.

## References

[1] Sutherland IW. The biofilm matrix–an immobilized but dynamic microbial environment. Trends in microbiology. 2001;9(5):222–7. 11336839 10.1016/s0966-842x(01)02012-1

[2] Flemming H-C, Neu TR, Wozniak DJ. The EPS matrix: the “house of biofilm cells”. Journal of Bacteriology. 2007;189(22):7945–7. 17675377 10.1128/JB.00858-07 PMC2168682

[3] Matsukawa M, Greenberg E. Putative exopolysaccharide synthesis genes influence Pseudomonas aeruginosa biofilm development. Journal of bacteriology. 2004;186(14):4449–56. 15231776 10.1128/JB.186.14.4449-4456.2004 PMC438629

[4] Flemming H-C, Leis A. Sorption Properties of Biofilms Encyclopedia of Environmental Microbiology: John Wiley & Sons, Inc.; 2003.

[5] Allesen‐Holm M, Barken KB, Yang L, Klausen M, Webb JS, Kjelleberg S, et al A characterization of DNA release in Pseudomonas aeruginosa cultures and biofilms. Molecular microbiology. 2006;59(4):1114–28. 16430688 10.1111/j.1365-2958.2005.05008.x

[6] Whitchurch CB, Tolker-Nielsen T, Ragas PC, Mattick JS. Extracellular DNA Required for Bacterial Biofilm Formation. Science. 2002;295(5559):1487 10.1126/science.295.5559.1487 11859186

[7] Wingender J, Jaeger K-E. Extracellular Enzymes in Biofilms Encyclopedia of Environmental Microbiology: John Wiley & Sons, Inc.; 2003.

[8] Geigl EM. On the circumstances surrounding the preservation and analysis of very old DNA. Archaeometry. 2002;44(3):337–42. 10.1111/1475-4754.t01-1-00066

[9] McBain AJ, Bartolo RG, Catrenich CE, Charbonneau D, Ledder RG, Rickard AH, et al Microbial Characterization of Biofilms in Domestic Drains and the Establishment of Stable Biofilm Microcosms. Appl Environ Microbiol. 2003;69(1):177–85. 10.1128/aem.69.1.177-185.2003 12513993 PMC152421

[10] Flores GE, Bates ST, Caporaso JG, Lauber CL, Leff JW, Knight R, et al Diversity, distribution and sources of bacteria in residential kitchens. Environmental Microbiology. 2012:n/a-n/a. 10.1111/1462-2920.12036 PMC5100818 23171378

[11] McBain AJ, Bartolo RG, Catrenich CE, Charbonneau D, Ledder RG, Price BB, et al Exposure of Sink Drain Microcosms to Triclosan: Population Dynamics and Antimicrobial Susceptibility. Appl Environ Microbiol. 2003;69(9):5433–42. 10.1128/aem.69.9.5433-5442.2003 12957932 PMC194980

[12] Butler JM, Shen Y, McCord BR. The development of reduced size STR amplicons as tools for analysis of degraded DNA. 2003;48(5):11. 14535668

[13] Pääbo S. Ancient DNA: extraction, characterization, molecular cloning, and enzymatic amplification. Proceedings of the National Academy of Sciences. 1989;86(6):1939–43. 10.1073/pnas.86.6.1939 PMC286820 2928314

[14] Simmon KE, Steadman DD, Durkin S, Baldwin A, Jeffrey WH, Sheridan P, et al Autoclave method for rapid preparation of bacterial PCR-template DNA. Journal of Microbiological Methods. 2004;56(2):143–9. 14744443 10.1016/j.mimet.2003.10.003

[15] Straub T, Baird C, Bartholomew RA, Colburn H, Seiner D, Victry K, et al Estimated copy number of Bacillus anthracis plasmids pXO1 and pXO2 using digital PCR. Journal of Microbiological Methods. 2013;92(1):9–10. 10.1016/j.mimet.2012.10.013 23142659

[16] Cardinale M, Brusetti L, Quatrini P, Borin S, Puglia AM, Rizzi A, et al Comparison of Different Primer Sets for Use in Automated Ribosomal Intergenic Spacer Analysis of Complex Bacterial Communities. Appl Environ Microbiol. 2004;70(10):6147–56. 10.1128/aem.70.10.6147-6156.2004 15466561 PMC522057

[17] Ramisse V, Patra G, Vaissaire J, Mock M. The Ba813 chromosomal DNA sequence effectively traces the whole Bacillus anthracis community. Journal of Applied Microbiology. 1999;87(2):224–8. 10.1046/j.1365-2672.1999.00874.x 10475954

[18] Volokhov D, Pomerantsev A, Kivovich V, Rasooly A, Chizhikov V. Identification of Bacillus anthracis by multiprobe microarray hybridization. Diagnostic Microbiology and Infectious Disease. 2004;49(3):163–71. 10.1016/j.diagmicrobio.2004.03.015 15246505

[19] Cheun HI, Makino SI, Watarai M, Shirahata T, Uchida I, Takeshi K. A simple and sensitive detection system for Bacillus anthracis in meat and tissue. Journal of Applied Microbiology. 2001;91(3):421–6. 10.1046/j.1365-2672.2001.01395.x 11556906

[20] Jackson PJ, Hugh-Jones ME, Adair DM, Green G, Hill KK, Kuske CR, et al PCR analysis of tissue samples from the 1979 Sverdlovsk anthrax victims: The presence of multiple Bacillus anthracis strains in different victims. Proceedings of the National Academy of Sciences. 1998;95(3):1224–9. 10.1073/pnas.95.3.1224 PMC18726 9448313

